# New sequence-based data on the relative DNA contents of chromosomes in the normal male and female human diploid genomes for radiation molecular cytogenetics

**DOI:** 10.1186/1755-8166-2-13

**Published:** 2009-06-05

**Authors:** Mikhail V Repin, Pavel I Golubev, Ludmila A Repina

**Affiliations:** 1 Dzhelepov Laboratory of Nuclear Problems, Joint Institute for Nuclear Research, Dubna, Moscow region, 141980, Russia; 2 Department of Physics, Lund University, Lund, S-221 00, Sweden; 3 Department of Radiation Safety of Human Spaceflights, State Research Center of the Russian Federation – Institute for Biomedical Problems, Russian Academy of Sciences, Moscow, 123007, Russia

## Abstract

**Background:**

The objective of this work is to obtain the correct relative DNA contents of chromosomes in the normal male and female human diploid genomes for the use at FISH analysis of radiation-induced chromosome aberrations.

**Results:**

The relative DNA contents of chromosomes in the male and female human diploid genomes have been calculated from the publicly available international Human Genome Project data. New sequence-based data on the relative DNA contents of human chromosomes were compared with the data recommended by the International Atomic Energy Agency in 2001. The differences in the values of the relative DNA contents of chromosomes obtained by using different approaches for 15 human chromosomes, mainly for large chromosomes, were below 2%. For the chromosomes 13, 17, 20 and 22 the differences were above 5%.

**Conclusion:**

New sequence-based data on the relative DNA contents of chromosomes in the normal male and female human diploid genomes were obtained. This approach, based on the genome sequence, can be recommended for the use in radiation molecular cytogenetics.

## Background

The fluorescence *in sit*u hybridization (FISH) technique [1,2] has facilitated rapid detection of stable chromosomal aberrations in human lymphocytes [3] and has become one of the widely used methods in radiation biodosimetry [4-6]. FISH analysis of radiation-induced chromosome aberrations (translocation analysis) was recommended by the International Atomic Energy Agency (IAEA) for estimating absorbed doses of ionizing radiation [7].

Several questions of radiation cytogenetics are connected with the comparison of results obtained by FISH analysis and those by conventional dicentric analysis [8-10] and with the intercomparison of results of FISH analysis with different DNA probes specific for individual chromosomes [11-13]. In this connection an approach based on the calculation of "genomic" frequencies of aberrations in dependence from the fraction of the diploid human genome covered by FISH probes was developed and corresponding equations were derived as early as 1992 [14].

It is necessary to know the fractions of the genome covered by FISH probes at the translocation analysis in order to obtain the whole genome equivalent genomic frequencies of chromosome aberrations [14]. In most cases whole chromosome FISH probes are used in radiation cytogenetics. Therefore, it is necessary to know the fractions of the human genome occupied by individual chromosomes.

The relative human DNA contents given in [7] are recommended by the IAEA for calculations of the genomic frequencies of radiation-induced aberrations. These values are derived from the data of Morton on the DNA contents of human chromosomes [15]. However, Morton's estimates of the DNA contents of individual human chromosomes are not exact because they are based on old experimental data obtained by different indirect physical methods [15] including autoradiography [16], image cytometry [17,18], flow cytometry [19-21].

With the increasing accuracy of chromosome aberration analysis [4], the importance of obtaining new exact and objective data on the absolute and relative DNA contents of human chromosomes for radiation cytogenetics is evident.

In the post-genomic era, with the completion of the international Human Genome Project [22], new more accurate data on the length of human chromosomes have been obtained. In this work the publicly available genome sequence data (the numbers of nucleotide base pairs) of the human chromosomes [23] are used for the calculation of relative DNA contents in the normal male and female diploid human genomes.

## Results and discussion

The total sizes of the normal male and female diploid human genomes and the relative DNA contents of chromosomes in the diploid genomes were calculated by using the Human Genome Project data on the chromosome lengths presented in the Ensembl database [23]. The results of these calculations are shown separately for the male and female diploid genomes in Table 1.

**Table 1:** Sequence-based DNA contents of the human chromosomes.

Chromosome	Chromosome length, bp	Relative DNA contents of chromosome pairs in diploid genome, %	
		
		Female	Male
1	247 249 719	8.1799	8.3135
2	242 951 149	8.0377	8.1690
3	199 501 827	6.6002	6.7080
4	191 273 063	6.3280	6.4313
5	180 857 866	5.9834	6.0811
6	170 899 992	5.6540	5.7463
7	158 821 424	5.2544	5.3402
8	146 274 826	4.8393	4.9183
9	140 273 252	4.6407	4.7165
10	135 374 737	4.4787	4.5518
11	134 452 384	4.4482	4.5208
12	132 349 534	4.3786	4.4501
13	114 142 980	3.7763	3.8379
14	106 368 585	3.5191	3.5765
15	100 338 915	3.3196	3.3738
16	88 827 254	2.9387	2.9867
17	78 774 742	2.6062	2.6487
18	76 117 153	2.5182	2.5594
19	63 811 651	2.1111	2.1456
20	62 435 964	2.0656	2.0993
21	46 944 323	1.5531	1.5785
22	49 691 432	1.6440	1.6708
X	154 913 754	5.1251	2.6044†
Y	57 772 954		0.9713†

The size of the diploid female genome	6 045 293 052	100	--

The size of the diploid male genome	5 948 152 252	--	100

The sequence-based absolute and relative DNA contents of the human chromosomes in the male and female diploid genomes calculated by using the international Human Genome Project data from Ensembl database, release 52 – December 2008 [23].

† Relative DNA contents of single X and single Y chromosomes in the male human diploid genome were calculated

**Figure 1:**
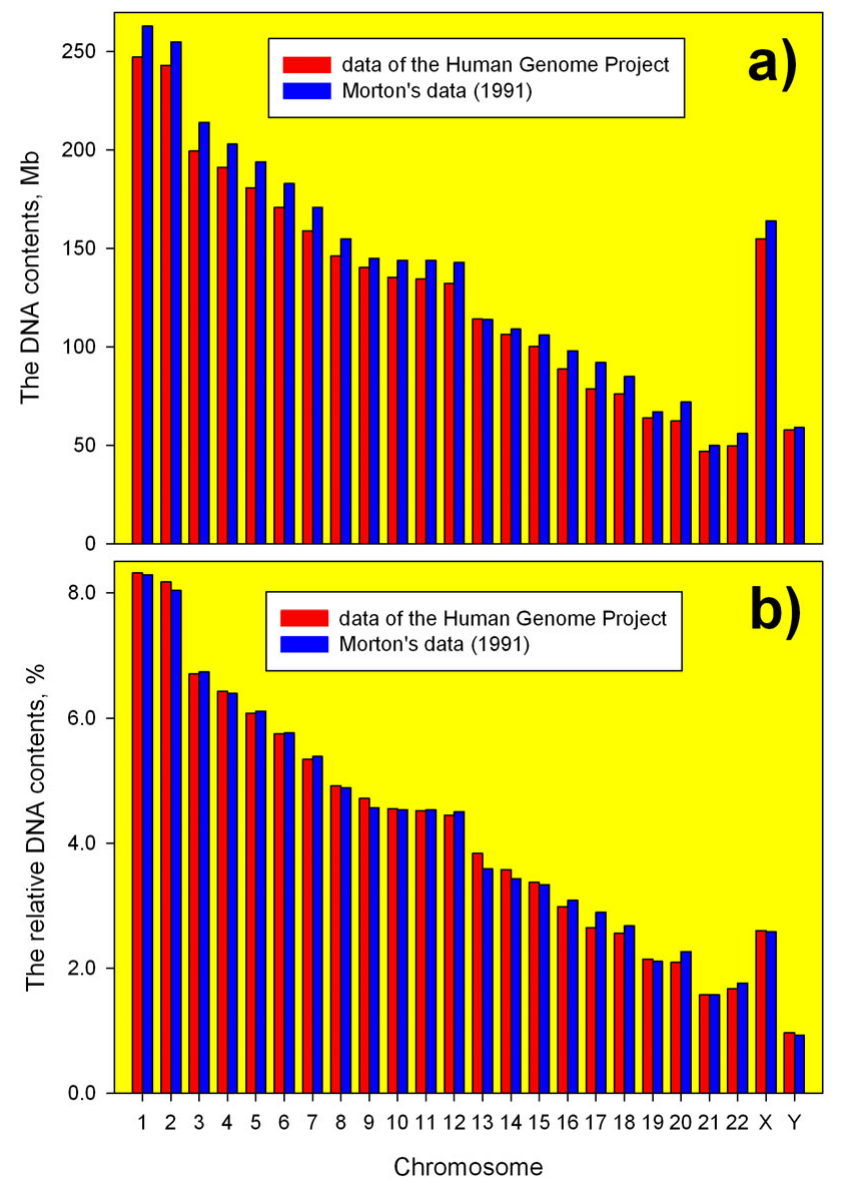
**The absolute (a) and relative (b) DNA contents of the human chromosomes obtained by different approaches**. **a)**. The absolute DNA contents of the human chromosomes according to the data of Morton [15] and the Human Genome Project data [23]. **b)**. The relative DNA contents of the chromosomes in the male human diploid genome from [7] and results of this work (see Table 1).

**Figure 2:**
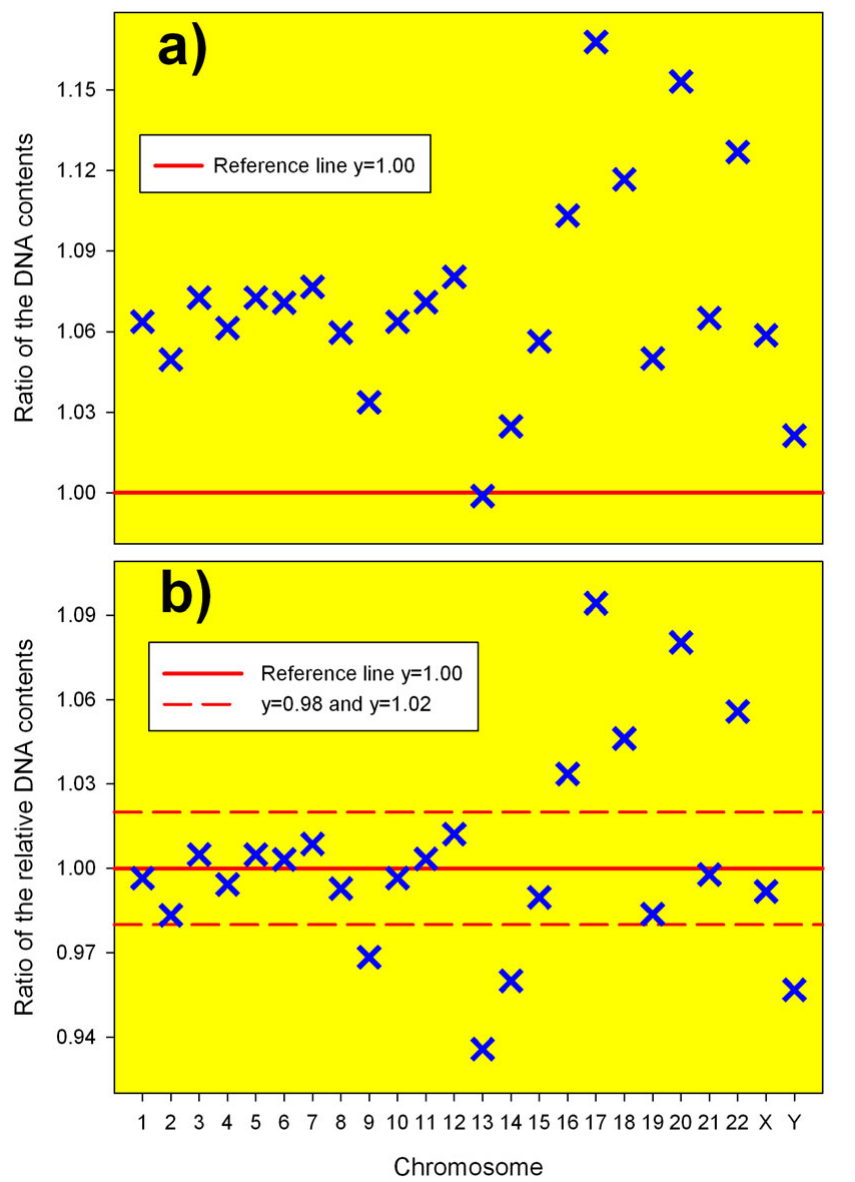
**Ratios of the absolute (a) and relative (b) DNA contents of the human chromosomes obtained by different approaches**. **a)**. Ratios between absolute DNA contents of the chromosomes in the male human diploid genome published by Morton [15] and Human Genome Project data [23]. **b)**. Ratios between relative DNA contents of the chromosomes in the male human diploid genome from [7] and results of this work (see Table 1).

The DNA contents of all chromosomes, except chromosome 13, were overestimated in the work of Morton [15] when compared with the Human Genome Project data (Figure 1a and 2a). The maximum difference in these estimates of the human chromosome lengths (~16%) was found for chromosome 17 (Figure 2a). The total sizes of the male and female human diploid genomes according to [15] (6 349 Mb and 6 454 Mb for the male and female, correspondingly) were overestimated approximately by 7% in comparison with the data presented in this work (Table 1).

The comparison of the data from Table 1 and the IAEA manual [7] has shown that the differences in the relative DNA contents of chromosomes in the normal human diploid genomes determined by different approaches are less than in their corresponding absolute DNA contents (Figures 2a and 2b). As it is seen from Figures 1b and 2b, the relative DNA contents of the human chromosomes in the male human diploid genome derived from the Ensembl's data are very close to those derived from Morton's data and recommended by the IAEA [7]. For 15 chromosomes, mainly for large chromosomes, the differences in their relative DNA contents in the human genomes obtained using different approaches are below 2% (Figure 2b).

However, noticeable differences (larger than 5%) were found in the relative DNA contents of chromosomes 13, 17, 20 and 22 in the human diploid genomes obtained by different approaches (Figure 2b). This result is explained by the poor accuracy of estimates of chromosome lengths by physical methods for small chromosomes than for larger ones. The difference in the relative DNA content of chromosome 17 derived from the human genome sequence data and from the estimates in the work [15] reaches the level of 9.4% (Figure 2b). Such large differences in the relative DNA contents of individual chromosomes obtained by different approaches could lead to different conclusions, in particular, about the radiosensitivity of these chromosomes and random or non-random distribution of radiation-induced damage in the human chromosomes.

The coefficient 2.05 in the formula of Lucas et al. [14] was re-calculated by using the new sequence-based chromosome lengths from Table 1. The recalculated coefficients for the male and female human genomes were equal to 2.0533 and 2.0528, respectively. These values are very close to the previously used value of 2.05.

Many radiobiological investigations were carried out with the use of DNA probes specific for large chromosomes because the probabilities of their damages by ionizing radiations and the levels of aberrations are highest and the translocation analysis is more effective. It should be noted that taking into account small differences in the values of the relative DNA contents of large chromosomes from work [7] and Table 1, general results and conclusions that were obtained in such investigations would be the same if the approach based on the genome sequence was used. Thus, in most cases the introduction of the correct data on the relative DNA contents of human chromosomes should not be complicated.

In spite of the high-quality sequencing data there are still some uncertainties about the gaps in the genome sequence and human genetic variations [22]. Recently, a considerable degree of genetic variations ranging to megabases in size was shown [24]. The 1000 Genomes project could provide a deeper understanding of human genetic variations [25].

Nevertheless, new values of the relative DNA contents of chromosomes in the normal human diploid genome based on the international Human Genome Project sequence data could be considered as the best data to date.

## Conclusion

At present we have the unique opportunity to use precise sequence-based parameters of the reference human genome including the relative DNA contents of chromosomes in the human genome instead of the approximate estimates that have been done by indirect methods at the initial stage of the Human Genome Project. New sequence-based data on the relative DNA contents of chromosomes in the normal male and female human diploid genomes were obtained. The approach, based on the DNA sequence data, can be recommended for the use in radiation molecular cytogenetics.

## Methods

The data on the lengths of each human chromosome were taken from the public Ensembl database , release 52 – December 2008 [23]. The sequence-based relative DNA contents of the male and female human diploid genomes occupied by each pair of autosome chromosomes were calculated (Table 1). Briefly, the total sizes of the male and female human diploid genomes were obtained by addition of the lengths of all 46 chromosomes: 22 pairs of the autosomes and two X chromosomes for the female genome and 22 pairs of the autosomes and two sex chromosomes X and Y for the male genome.

For each pair of the autosomes the relative DNA contents were calculated as a ratio of the doubled DNA size to the size of diploid female and male genomes, correspondingly. Similarly the relative DNA content of the sex chromosome X in the female genome was calculated. The single DNA contents in the genome were used to obtain the relative DNA content of the sex chromosomes in the diploid male human genome.

In the formula derived by Lucas et al. [14]*F*_*p *_= 2.05 *f*_*p*_(1 - *f*_*p*_)*F*_*G*_, relating the translocation frequency, *F*_*p*_, measured using FISH to the genomic translocation frequency, *F*_*G*_, where *f*_*p *_is the fraction of the genome covered by the composite probe, the coefficient 2.05 was recalculated separately for the human female and male genomes by using the sequence-based relative DNA contents of the chromosomes from Table 1:



where *C*_*i *_is a fraction of the DNA content of the *i*-chromosome in the male or female human diploid genome.

## Competing interests

The authors declare that they have no competing interests.

## Authors' contributions

MVR wrote the manuscript and PIG and LAR contributed significant editorial input and original ideas. All authors read and approved the final manuscript.

## References

[B1] Pinkel D, Straume T, Gray JW (1986). Cytogenetic analysis using quantitative, high-sensitivity, fluorescence hybridization. Proc Natl Acad Sci USA.

[B2] Pinkel D, Landegent J, Collins C, Fuscoe J, Segraves R, Lucas J, Gray J (1988). Fluorescence *in situ *hybridization with human chromosome-specific libraries: Detection of trisomy 21 and translocations of chromosome 4. Proc Natl Acad Sci USA.

[B3] Lucas JN, Tenjin T, Straume T, Pinkel D, Moore D, Litt M, Gray JW (1989). Rapid human chromosome aberration analysis using fluorescence *in situ *hybridization. Int J Radiat Biol.

[B4] Kanda R (2000). Improvement of accuracy of chromosome aberration analysis for biological radiation dosimetry. J Radiat Res (Tokyo).

[B5] Tucker JD (2001). FISH cytogenetics and the future of radiation biodosimetry. Radiat Prot Dosimetry.

[B6] Edwards AA, Lindholm C, Darroudi F, Stephan G, Romm H, Barquinero J, Barrios L, Caballin MR, Roy L, Whitehouse CA (2005). Review of translocations detected by FISH for retrospective biological dosimetry applications. Radiat Prot Dosimetry.

[B7] IAEA (2001). Cytogenetic analysis for radiation dose assessment: a manual.

[B8] Kanda R, Hayata I (1996). Comparison of the yields of translocations and dicentrics measured using conventional Giemsa staining and chromosome painting. Int J Radiat Biol.

[B9] Lindholm C, Luomahaara S, Koivistoinen A, Ilus T, Edwards AA, Salomaa S (1998). Comparison of dose-response curves for chromosomal aberrations established by chromosome painting and conventional analysis. Int J Radiat Biol.

[B10] Nakano M, Kodama Y, Ohtaki K, Itoh M, Delongchamp R, Awa AA, Nakamura N (2001). Detection of stable chromosome aberrations by FISH in A-bomb survivors: comparison with previous solid Giemsa staining data on the same 230 individuals. Int J Radiat Biol.

[B11] Braselmann H, Kulka U, Huber R, Figel HM, Zitzelsberger H (2003). Distribution of radiation-induced exchange aberrations in all human chromosomes. Int J Radiat Biol.

[B12] Anderson RM, Sumption ND, Papworth DG, Goodhead DT (2006). Chromosome breakpoint distribution of damage induced in peripheral blood lymphocytes by densely ionizing radiation. Int J Radiat Biol.

[B13] Lindholm C, Romm H, Stephan G, Schmid E, Moquet J, Edwards A (2002). Intercomparison of translocation and dicentric frequencies between laboratories in a follow-up of the radiological accident in Estonia. Int J Radiat Biol.

[B14] Lucas JN, Awa A, Straume T, Poggensee M, Kodama Y, Nakano M, Ohtaki K, Weier H-U, Pinkel D, Gray J (1992). Rapid translocation frequency analysis in humans decades after exposure to ionizing radiation. Int J Radiat Biol.

[B15] Morton NE (1991). Parameters of the human genome. Proc Natl Acad Sci USA.

[B16] Korenberg JR, Engels WR (1978). Base ratio, DNA content, and quinacrine-brightness of human chromosomes. Proc Natl Acad Sci USA.

[B17] Mayall BH, Carrano AV, Moore DH, Ashworth LK, Bennett DE, Mendelsohn ML (1984). The DNA-based human karyotype. Cytometry.

[B18] Mendelsohn ML, Mayall BH, Bogart E, Moore DH, Perry BH (1973). DNA content and DNA-based centromeric index of the 24 human chromosomes. Science.

[B19] Harris P, Boyd E, Young BD, Ferguson-Smith MA (1986). Determination of the DNA content of human chromosomes by flow cytometry. Cytogenet Cell Genet.

[B20] Langlois RG, Yu L-C, Gray JW, Carrano AV (1982). Quantitative karyotyping of human chromosomes by dual beam flow cytometry. Proc Natl Acad Sci USA.

[B21] Tiersch TR, Chandler RW, Wachtel SS, Elias S (1989). Reference standards for flow cytometry and application in comparative studies of nuclear DNA content. Cytometry.

[B22] International Human Genome Sequencing Consortium (2004). Finishing the euchromatic sequence of the human genome. Nature.

[B23] Hubbard TJ, Aken BL, Ayling S, Ballester B, Beal K, Bragin E, Brent S, Chen Y, Clapham P, Clarke L (2009). Ensembl 2009. Nucleic Acids Res.

[B24] Sebat J, Lakshmi B, Troge J, Alexander J, Young J, Lundin P, Maner S, Massa H, Walker M, Chi M (2004). Large-scale copy number polymorphism in the human genome. Science.

[B25] Siva N (2008). 1000 Genomes project. Nat Biotechnol.

